# Association between Nutritional Status and Positive Childhood Disability Screening Using the Ten Questions Plus Tool in Sarlahi, Nepal

**DOI:** 10.3329/jhpn.v28i6.6607

**Published:** 2010-12

**Authors:** L. Wu, J. Katz, L.C. Mullany, E. Haytmanek, S.K. Khatry, G.L. Darmstadt, K.P. West, S.C. LeClerq, J.M. Tielsch

**Affiliations:** ^1^ Department of International Health, Bloomberg School of Public Health, Johns Hopkins University, Baltimore, MD, USA; ^2^ Institute of Medicine, Washington, DC, USA; ^3^ Nepal Nutrition Intervention Project–Sarlahi, Kathmandu, Nepal; ^4^ Integrated Health Solutions Development, Global Health Programme, Bill & Melinda Gates Foundation, Seattle, Washington, DC, USA

**Keywords:** Child, Child development, Child nutrition, Cognitive development, Disability, Stunting, Nepal

## Abstract

The study was conducted to examine the association between the indicators of malnutrition and disability of children as reported by caregivers. The Ten Questions Plus questionnaire was administered to caregivers of 1,902 children aged 1–9 years, during August 2007–March 2008, in rural Nepal. Height and weight of children were also measured. The main outcome was a positive response to one or more questions. In total, 514 (27%) children had a positive response to at least one question. Moderate stunting [odds ratio (OR)=1.47, 95% confidence interval (CI) 1.02–2.12) and severe (OR=2.39, 95% CI 1.60–3.57) stunting were independently associated with reported delay in sitting, standing, or walking. Severe stunting was also associated with report of delayed learning compared to other children of similar age (OR=2.01, 95% CI 1.27–3.20). Parental report of disability was quite prevalent in this setting, with over a quarter of the sample screening positive. Chronic malnutrition may be associated with delayed motor and mental development.

## INTRODUCTION

Disability is defined by the United Nations (UN) as “long-term physical, mental, intellectual or sensory impairments which, in interaction with various barriers, may hinder [a person's] full and effective participation in society on an equal basis with others” (1). With the adoption of the UN Convention on the Rights of Persons with Disabilities, the United Nations Children's Fund (UNICEF) has made the inclusion and development of children with disabilities a priority issue, and the World Health Organization (WHO) estimates that over 200 million children worldwide are at risk for not meeting their developmental potential (2). Few data are available, however, on the prevalence of and risk factors for children with disabilities in developing countries.

The Ten Questions (TQ) screen was developed in 1984 as a tool for screening children aged 2–9 years in resource-poor settings to identify disabilities and to refer screen-positive children for further clinical evaluation and intervention (3). It focuses on questions about general functional abilities and milestones rather than culture-specific skills (4). A child is considered screen-positive if his or her parent responds ‘yes’ to one or more of the 10 questions. The TQ tool, evaluated and validated in over 20 countries to date, is highly sensitive (5–7). The tool has a low positive predictive value for serious disability but these ‘false-positive’ children are often found to have either mild disability or a health condition requiring treatment (5). Thus, the TQ tool is best used as a screen for identifying children with developmental delays or who are at an increased risk of disability (4).

The UNICEF currently recommends the incorporation of the TQ tool into its Multiple Indicator Cluster Survey (MICS) programme to address the gap in knowledge about childhood disabilities in developing countries. The most recent MICS in 2005–2006 included results of TQ screening of over 190,000 children in 18 developing countries. Nutritional factors, such as not being breastfed, no receipt of vitamin A supplementation, wasting, and stunting, were significantly associated with screening positive for disability (8). In their data, anthropometric measures were only available for children aged less than five years, and little is known about the association of poor nutritional status with loss of developmental potential in children aged over five years (2, 4, 8). This age-group is important to measure because child development is related to school attendance, which then affects earning potential later in life (2).

In the present study, we used data from rural southern Nepal to estimate the prevalence of parental report of various types of disabilities in children aged 1–9 years and their associations with nutritional status using a modified TQ—the Ten Questions Plus (TQP). While the TQ tool has been used in Bangladesh, Pakistan, and India, it has not been used in Nepal (5, 9). Additionally, most prior evaluation of TQ has been done in a mix of urban and rural areas, and our community-based sample comes from an entirely rural area. The additional eleventh question (the ‘Plus’) has not been previously evaluated in the published literature. Lastly, while the MICS looked at the overall prevalence of screen-positivity, we also examined the association of nutritional indicators with individual questions on the screen. This can provide additional information on specific types of disability and the burden in this rural South Asian population, adding to the body of knowledge on where to target interventions in similar settings to prevent or treat childhood disability (4).

## MATERIALS AND METHODS

### Ten Questions Plus questionnaire

The TQ tool, with one question added to the original 10, was developed and tested by the Bangladesh Protibondhi Foundation as part of the International Pilot Study of Severe Childhood Disability (10). The additional eleventh question assesses behavioural problems, e.g. frequent tantrums, aggressive behaviour, or difficulty relating to people, and the resulting screening tool is referred to as the TQ Plus (TQP) questionnaire (questions in Table 1).

**Table 1.** Ten questions plus questionnaire responses (n=1,902)

Question	No.*	Yes (%)
Child's mother	1,322	69.5
Child's father	102	5.4
Other relatives (grandparent, aunt/uncle, sibling, other)	473	24.9
Q1 Motor milestones		
Compared with other children, did your child have any serious delay in sitting, standing, or walking?	196	10.3
Q2 Vision		
Compared with other children, does your child have difficulty in seeing, either in daytime or night time?	16	0.8
Q3 Hearing		
Does your child appear to have difficulty in hearing?	43	2.3
Q4 Understanding		
When you tell your child to do something, does s/he have problems in understanding what you are saying?	37	2.0
Q5 Movement		
Does your child have difficulty in walking or moving his/her arms or does s/he have weakness and/or stiffness in the arms or legs?	31	1.6
Q6 Fits/seizures		
Does your child have fits/convulsions, become rigid or lose consciousness?	28	1.5
Q7 Learning		
Does the child have trouble in learning to do things like other children of his/her age?	182	9.6
Q8 No speech		
Is your child mute (not able to speak at all)?	46	2.4
Q9 Speech**		
Is your child's speech in any way different from normal?	58	3.7
Q10 Slowness**		
Compared with other children of his/her age, does your child appear in any way mentally slow, dull, or backward?	45	2.9
Q11 Behavioural problems**		
Does your child show any behavioural problem, such as frequent tantrums, aggressive behaviour, or difficulty in relating to people?	70	4.5
Summary		
Positive screening for disability (‘yes’ on any of the above questions)	514	27.0

*Missing values: Q1: 4, Q2: 2, Q3: 3, Q4: 1, Q6: 6, Q7: 8, Q8: 3, Q9: 2, Q10: 3, and Q11: 1;

**Only asked of children aged 3 years or older (n=1,559)

### Study population and data collection

The TQP was administered to a sample of children who had previously participated in the Nepal Nutrition Intervention Project–Sarlahi (NNIPS-4) study, which has been described in detail elsewhere (11, 12). Given the prevalence of a positive screen and of stunting in this population, the sample-size was adequate to detect an odds ratio of 1.4 with power of 90%. Briefly, the NNIPS-4 was a community-based, cluster-randomized 2×2 factorial trial of the impact of daily iron and/or zinc supplementation on mortality, morbidity, and physical growth. Children were cluster-randomized to receive one of four supplements (placebo, iron/folic acid, zinc, or zinc and iron/folic acid) daily from the time of enrollment (1 month) to discharge (36 months). The trial was conducted in Sarlahi district during October 2001–January 2006 among 426 clusters (sectors) across 30 Village Development Committees (VDCs), a government-defined administrative unit.

The sample for the TQP screening and nutrition study included children residing in one (Ishwarpur) of the 30 VDCs and who had previously participated in the NNIPS-4 trial. The TQP screening was nested within a larger follow-up of anthropometric status among infants in the previous iron/zinc trial (13). The additional TQP data-collection component was begun after 30% of the larger study had been completed, and all eligible children not yet visited were included after the starting date. The instrument was translated from English into Nepali and then backtranslated by a different person to make sure that the English and Nepali versions were the same. There were seven interviewers, one of whom conducted 30% of the interviews, two conducted 18% each, and the remainder conducted 10%, except for one who conducted 4% of the interviews. We do not have inter- and intra-reliability on these interviewers. The questionnaire was pretested in a population similar to this one but who were not participants in this follow-up trial. Once collection of data using the TQP started on 18 September 2007, anthropometric measures were taken at the same visit as the TQP administration. Data were collected through March 2008. Although this is not a random sample of NNIPS-4 participants, the children selected for interview represent a geographically-distributed population throughout Ishwarpur.

The standing height of the child was measured using a portable Harpenden stadiometer to the nearest 0.1 cm. Weight was measured using the Seca 881u scale to the nearest 0.1 kg. Mid-upper arm circumference (MUAC) was measured to the nearest 0.1 cm using an insertion tape at the midpoint of the upper left arm with the left sleeve of the child removed. The tape was loosened and repositioned in between each reading. Height, weight, and MUAC were measured three times each, and the median value of the three measures was used for analysis. Approximately 1 in 30 measurements were repeated to check inter- and intra-observer reliability.

### Statistics

A summary indicator variable for positive disability screening was defined as a positive response on one or more of the screening questions (4). Weight-for-age z-score (WAZ), height-for-age z-score (HAZ), weight-for-height z-score (WHZ), and body mass index-for-age z-score (BMIZ) were calculated using the WHO 2005 reference population (14). The WHO 2000 reference population was used if the age-range of the child did not fall within the age range of the 2005 reference population. WAZ, HAZ, WHZ, and BMIZ were also dichotomized as <−2 standard deviation (SD) from the mean of the reference population and less than −3 SD. Children with WHZ or BMIZ <−2 SD indicate moderate wasting/acute malnutrition, and those with WHZ or BMIZ <−3 SD indicate severe wasting/acute malnutrition. Likewise, children with HAZ <−2 SD indicate moderate stunting/chronic malnutrition, and those with HAZ <−3 SD indicate severe stunting.

We modelled the adjusted odds of a positive response to any question as a function of each of the dichotomized z-scores (WAZ <−2 SD, HAZ <−2 SD, WHZ <−2 SD, BMIZ <−2 SD, WAZ <−3 SD, HAZ <−3 SD, WHZ <−3 SD, and BMIZ <−3 SD). We also used multivariate regression to model the odds of a positive disability screen for a combination of anthropometric measures (keeping −2 SD variables and −3 SD variables together). BMIZ and WHZ were never modelled together in the logistic regressions because these are highly correlated and are both indicators of wasting. We also modelled the odds of a positive response to each of the 11 questions separately using the same procedure outlined above. In all the logistic regression models, we adjusted for the age and sex of the child, maternal literacy, household caste, ethnicity, iron and zinc supplementation group, and an asset ownership scale (an ordinal variable ranging from 0 to 10 from a composite score of 1 if ‘yes’, 0 if ‘no’ on ownership of latrines, cattle, goats, carts, bicycles, radios, electricity in the home, land, televisions, and presence of servants) (15).

We performed likelihood ratio tests to compare extended models to null models not adjusted for potential confounders. Interactions of age and sex with anthropometric measures were tested in logistic regression models. We examined the number of unique covariate patterns in the data and assessed goodness-of-fit of the model using Pearson's method because there were many non-unique covariate patterns. Last, we looked at model residuals, leverage, and influence to check model fit and assumptions. Data were analyzed using the Stata software (version 10) and the SAS software (version 9.2) (16, 17).

### Ethics

Verbal informed consent was obtained from the primary caregiver of each child. The Ethical Review Board of the Nepal Health Research Council, Kathmandu, and the Institutional Review Board of the Johns Hopkins Bloomberg School of Public Health approved the study.

## RESULTS

In total, 3,194 children from Ishwarpur in the original NNIPS-4 iron/zinc supplementation trial were eligible to receive the TQP screen (Fig.). Twenty-eight children had died, 59 had moved, and one refused to participate. Of the remaining 3,106 children, the TQP was administered to a population-based sample of 1,902. Of them, 1,728 (91%) were weighed, 1,651 (87%) had height, and 1,735 (91%) had MUAC measured. Age and sex were available for all the 1,902 children.

**Fig.**
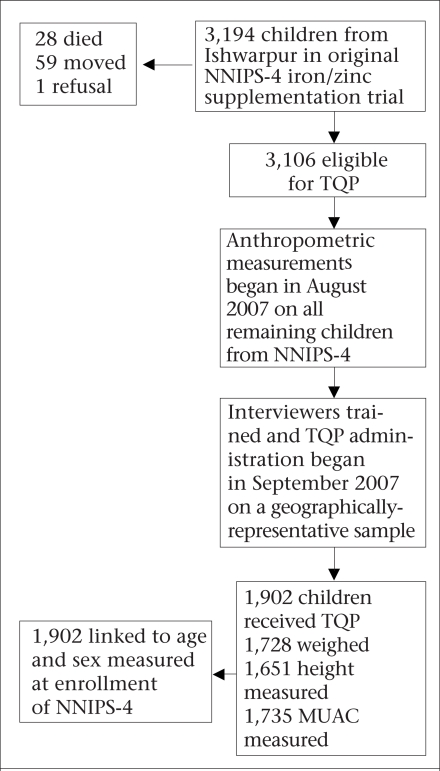
Study profile

MUAC=Mid-upper arm circumference;
NNIPS=Nepal Nutrition Intervention Project–Sarlahi;
TQP=Ten Questions Plus

The eligible children from Ishwarpur, who did not receive the TQP screen (n=1,251), were comparable on all characteristics except that those who received the screen had a higher prevalence of wasting as measured by WHZ (38.2% vs 29.9%) and BMIZ (31.4% vs 21.5%). The children who received the TQP came from families that owned less land (46.6% vs 54.4%, p<0.00001) and fewer bullock carts (9.6% vs 16.0%, p<0.00001) than those who did not receive the TQP.

The age of the TQP participants ranged from 1.4 to 8.9 years (mean 5.0 years); 51.4% were male (Table 2). The prevalence of WHZ <−2 and <−3 was 38.2% and 14.3% respectively. The prevalence of HAZ <−2 and −3 was 49.5% and 17.1% respectively. The mean BMI was 14.0 (SD=1.4), and the mean MUAC was 14.6 cm (SD=1.1). The majority of the children came from the *Vaiysha* (farmers, traders, craftsmen) caste (58.2%); smaller proportions were from the *Shudra* (menial, servants, craftsmen) castes (13.6%); and 21.1% were from Muslim families. Eighty-five percent belonged to the *Madheshi* ethnic group (native to Nepal and residing in the southern plains). About 20% of the mothers (n=363) were literate. Less than 10% of the households owned a latrine (n=160), servant (n=83), or bullock cart (n=183).

**Table 2.** Characteristics of study population

Characteristics	Mean±SD or No. (%)
Child characteristics	
Age (years)	5.0±1.9
Male	978 (51.4)
Female	924±48.6
Mid-upper arm circumference (cm)	14.6±1.1
Body mass index (kg/m^2^)	14.0±1.4
WAZ*	−2.6±1.2
WAZ <−2 SD	1,136 (69.7)
WAZ <−3 SD	541 (33.2)
HAZ*	−2.0±1.2
HAZ <−2 SD	769 (49.5)
HAZ <−3 SD	265 (17.1)
WHZ*	−1.8±1.4
WHZ <−2 SD	571 (38.2)
WHZ <−3 SD	214 (14.3)
BMIZ*	−1.6±1.5
BMIZ <−2 SD	487 (31.4)
BMIZ <−3 SD	206 (13.3)
Household characteristics	
Caste	
Brahmin	87 (4.6)
Chhetri	48 (2.5)
Vaiysha	1,105 (58.2)
Shudra	258 (13.6)
Muslim	400 (21.1)
Ethnicity	
Pahadi	277 (14.7)
Madheshi	1,607 (85.3)
Mother literate	363 (19.9)
Father literate	1,037 (55.1)
Latrine in house	160 (8.4)
Servant(s) in house	83 (4.4)
Cattle	994 (52.4)
Goat(s)	758 (39.9)
Land ownership	873 (46.6)
Bullock cart(s)	183 (9.6)
Bicycle(s)	954 (50.3)
Radio(s)	440 (23.1)
Electricity in home	536 (28.2)
Television	388 (20.5)

*Combination of WHO 2000 and 2005 reference populations, each of which covers different age range (14); 2000 reference population was used only if not in age range for 2005 reference population; Missing values: Weight: 174, Height: 251, MUAC: 167, BMI: 253, WAZ: 273, HAZ: 349, WHZ: 401, BMIZ: 351, Caste: 4, Ethnicity: 18, Maternal literacy: 73, Paternal literacy: 21, Latrine: 5, Servants: 3, Cattle: 3, Goats: 3, Land: 29, Carts: 4, Bicycles: 5, Radios: 4, Electricity: 3, Televisions:8;

BMIZ=BMI-for-age z-score;

HAZ=Height-for-age z-score;

MUAC=Mid-upper arm circumference;

SD=Standard deviation from the mean;

WAZ=Weight-for-age z-score;

WHO=World Health Organization;

WHZ=Weight-for-height z-score

Of the respondents, 69.5% were mothers (n=1,322), 5.4% were fathers (n=102), or 25.1% were other relatives (n=473). The proportion of positive responses (of 1,902) to each question (Table 1) ranged from 0.8% (n=16) answering ‘yes’ to Question 2: vision problems to 10.3% (n=196) answering ‘yes’ to Question 1: motor milestone delay.

Overall, 514 (27%) children screened positive for disability, with a ‘yes’ response to at least one question. Of these positive responses, 339 (64.4%) screened positive based on two questions (Question 1: motor milestones and Question 7: learning). Severe stunting was marginally associated with an increased adjusted odds of screening positive (OR=1.33, 95% CI 0.98–1.82).

Sex and continuous age (years) were independently significantly associated with positive TQP screening (OR female=0.77, 95% CI 0.65–0.97; OR age=0.82, 95% CI 0.78–0.87). For individual questions, we examined the association with nutritional indicators for those questions where 3% or more of the respondents answered positively (Table 3). The prevalence of a positive TQP screen was 31.2% among the severely-stunted children and 24.3% among the severely-underweight children.

**Table 3.** Results of logistic regression analyses with TQP outcomes of interest

Outcome severity of malnutrition	Wasting‡				Stunting‡			
	Crude		Adjusted*		Crude		Adjusted**	
	OR (95% CI)	p value	OR (95% CI)	p value	OR (95% CI)	p value	OR (95% CI)	p value
Positive screen								
Moderate†	0.94 (0.74–1.19)	0.60	0.96 (0.75–1.24)	0.72	1.14 (0.90–1.43)	0.27	1.04 (0.81–1.34)	0.76
Severe†	0.86 (0.62–1.21)	0.39	0.94 (0.66–1.35)	0.74	1.51 (1.13–2.00)	0.005	1.33 (0.98–1.82)	0.07
Q1: Motor milestones§								
Moderate	1.17 (0.84–1.64)	0.34	1.17 (0.82–1.66)	0.39	1.55 (1.11–2.16)	0.01	1.47 (1.02–2.12)	0.04
Severe	1.29 (0.82–2.01)	0.27	1.41 (0.88–2.25)	0.15	2.28 (1.58–3.29)	<0.0001	2.39 (1.60–3.57)	<0.0001
Q7: Learning§								
Moderate	0.83 (0.57–1.21)	0.33	0.83 (0.54–1.27)	0.38	1.75 (1.22–2.52)	0.003	1.50 (0.96–2.34)	0.07
Severe	0.62 (0.34–1.13)	0.12	0.81 (0.41–1.60)	0.54	2.84 (1.94–4.15)	<0.0001	2.01 (1.27–3.20)	0.003
Q9: Speech§								
Moderate	0.84 (0.45–1.56)	0.58	0.75 (0.39–1.44)	0.38	0.81 (0.45–1.46)	0.48	0.75 (0.39–1.44)	0.58
Severe	1.38 (0.65–2.90)	0.40	1.25 (0.56–2.78)	0.59	0.52 (0.18–1.46)	0.21	0.48 (0.17–1.39)	0.18
Q11: Behavioural problems§								
Moderate	0.90 (0.52–1.55)	0.70	0.93 (0.52–1.67)	0.81	1.17 (0.69–1.97)	0.56	1.22 (0.69–2.16)	0.50
Severe	0.75 (0.34–1.68)	0.49	0.85 (0.37–1.94)	0.70	0.73 (0.33–1.63)	0.44	0.68 (0.28–1.65)	0.39

*Logistic regression models adjusted for stunting, age (years) and sex of the child, maternal literacy (yes/no), household caste, ethnicity, iron/zinc supplementation group, and an asset ownership scale (ordinal variable ranging from 0 to 10 from a composite score of 1 if ‘yes’, 0 if ‘no’ on ownership of latrines, cattle, goats, carts, bicycles, radios, electricity in home, land, televisions, and presence of servants);

**Logistic regression models adjusted for wasting, age (years) and sex of the child, maternal literacy (yes/no), household caste, ethnicity, iron/zinc supplementation group, and an asset ownership scale (ordinal variable ranging from 0 to 10 from a composite score of 1 if ‘yes’, 0 if ‘no’ on ownership of latrines, cattle, goats, carts, bicycles, radios, electricity in home, land, televisions, and presence of servants);

§Refer to Table 1 for full questions;

†Moderate defined as <−2 SD and severe defined as <−3 SD;

‡Wasting defined as WHZ <−2 SD, and stunting defined as HAZ <−2 SD;

CI=Confidence interval;

HAZ=Height-for-age z-score;

OR=Odds ratio;

SD=Standard deviation from the mean;

TQP=Ten Questions Plus;

WHZ=Weight-for-height z-score

Moderate stunting (OR=1.47, 95% CI 1.02–2.12) and severe stunting (OR=2.39, 95% CI 1.60–3.57) were each associated with increased adjusted odds of a positive response to Question 1: motor milestone delay. Severe stunting was also associated with increased adjusted odds of a positive response to Question 7: learning (OR=2.01, 95% CI 1.27–3.20). The p values for likelihood ratio tests for each model were all <0.0001. The p values from Hosmer-Lemeshow goodness-of-fit tests for each model were <0.05.

## DISCUSSION

Overall, roughly one in four children in our sample screened positive on the TQP. Moderate stunting and severe stunting were associated with parental report of delayed motor milestone attainment for sitting, standing, and walking. Stunting was also associated with reports of delayed mental development, such as not learning to do things like other children of the same age. The two most common positive responses were associated with relative motor and cognitive deficits, as perceived by parental report.

The association between positive screening on the TQ and stunting has also been found in South America, Africa, eastern Europe, and Asia (see footnote in Table 4) (2). A validation study of the TQ tool in Pakistan estimated that the screen alone overestimated the prevalence of true child disability by more than 300% (18). Our TQP screen-positive proportion of 27.0% is a reasonable estimate of those children who are at an increased risk of disability or developmental delay given the global range of screen-positive proportions (Table 4). Results of another study in Pakistan with both urban and rural children, using the TQ to screen for mental retardation, showed that rural children were at greater risk of mental disability than urban children (19). We found a higher proportion of screen-positive cases in our rural sample than was found in Bangladesh, which included rural and urban areas (20.9% screen-positive) (Table 4) (4).

**Table 4.** Comparisons of TQ results across countries

Outcome	MICS (4)				
	Low (country)	High (country)	Nepal rural	Bangladesh urban and rural (4)	Pakistan mostly urban (19)
Positive screen (%)					
All children	2.0 (Uzbekistan)	31.0 (CAR)	27.0*	20.9	14.7
In severely-stunted children	6.1 (Uzbekistan)	55.6 (Belize)	31.2*	NA	NA
In severely underweight children	0.0 (Albania and Uzbekistan)	100.0 (Montenegro)	24.3*	NA	NA
Yes (%)					(18)
Motor milestones	0.4 (Uzbekistan and Montenegro)	8.6 (Belize)	10.3	7.4	4.5
Fits/seizures	0.3 (Uzbekistan)	7.1 (Thailand)	1.5	1.8	2.8
Learning	0.2 (Montenegro)	11.8 (CAR)	9.6	5.0	0.9
Behavioural problems	NA	NA	4.5	0.6†	NA

*Limited to TQ only (excludes Q11 on behavioural problems);

†After clinical confirmation; % of children with positive screen found to be associated with increasing severity of stunting in Belize, Bosnia and Herzegovina, Cameroon, Ghana, Mauritania, Mongolia, Montenegro, Sao Tome and Principe, Serbia, Sierra Leone, Suriname, TYFR Macedonia, and Uzbekistan (4);

CAR=Central African Republic;

MICS=Multiple indicator cluster survey;

NA=Not available;

TQ=Ten Questions

We have previously studied a subset (n=485) of the 1,902 children in Sarlahi, Nepal and found that children with better length-for-age and weight-for-length z-scores were associated with earlier age at walking (20). However, the TQP screen has never been used in this population before the present study was conducted. The present study followed children at a later age than our previous study, after most of them have attained their major motor milestones. Additionally, the TQP can identify multiple disabilities as it screens for many other physical and cognitive disabilities beyond motor milestones.

In our study, males had a higher probability of screening positive (OR=1.30), similar to that found in Bangladesh (OR=1.25) and Pakistan (OR=1.32) (4, 19). Our findings of a 10.3% positive response to the developmental milestone question and a 9.6% positive response to the learning question are higher than that found in any of the 20 countries evaluated by the UNICEF (Table 4). The differences between our results and those of other countries may be attributable to cultural variations in translation and understanding of the meaning of the questions, which should be investigated further across South Asia. However, evaluation of the reliability of the TQ in countries with different cultures and levels of socioeconomic development has validated the cross-cultural applicability of the screen (21). Thus, the differences we found may indicate a true higher prevalence of disability relating to motor development and learning problems in this area of rural Nepal than in other southeast Asian countries.

The eleventh question on behavioural problems was more recently added to the questionnaire and has not yet been validated in multiple countries. Where it was evaluated in Bangladesh, there was a 0.6% prevalence of positive response to this question, which differs from 4.4% prevalence we found in our sample (Ferdous S *et al*. Prevalence of childhood disability: the TQP study in Bangladesh. Presented at International Association for the Scientific Study of Intellectual Disabilities Conference, 18 June 2004, Montpellier, France). However, it should be noted that 0.6% prevalence was observed after clinical confirmation was made on those who initially screened positive; so, it is difficult to compare our findings with clinically-confirmed behavioural problems. This question was only asked about children aged three years and older but the percent responding positively to this question did not vary by age of the child. Since this is a new question, it will likely need validation in other settings.

The underlying biological mechanisms for childhood mental and physical disability are not completely understood and are likely multi-factorial. To date, a causal relationship between stunting and risk of disability could not be established but evidence from a global review supports that malnutrition impairs motor and mental development (4). Additionally, results of studies on children aged less than two years in Zanzibar indicate that nutritional deficiency contributes to cognitive and physical developmental delays (22, 23). Chronic malnutrition may contribute to an increased risk of mental developmental delays, or it may be that developmentally-delayed children do not eat as well or receive the same amount of food as those not considered developmentally delayed by their parents (24–26).

Further research needs to assess what roles breastfeeding and micronutrient status play in the biological process of disability development (4, 8). In this population, infants were almost universally but rarely exclusively breastfed. Additionally, there are variations in the timing of initiation, which may affect developmental processes later in life (27).

Strengths of our study include that it is population-based with a large sample-size, and the children screened for disability represent a geographically-distributed sample of Ishwarpur in a rural area of Nepal. Prior parental reports on disability in South Asia have not included the question on behavioural concerns, which were shown to be quite prevalent in this setting. While the association between stunting and positive screening was also found in the results of the MICS 3, anthropometric variables were only available in children aged 2–4 years (8).

### Limitations

Limitations of the study include that the sample was not necessarily representative of the population of Sarlahi or Nepal. It comprised a cohort of survivors of a population-based micronutrient trial. Although we found that the participants were slightly more wasted and may be less socioeconomically well-off compared to those children who did not receive the TQP, we do not believe that this affects the association we found between stunting and screen-positivity. Since we were not able to conduct definitive neuro-developmental evaluations of the children who screened positive, we do not know the sensitivity and specificity of the tool in a rural population like that in Nepal, although its specificity has been demonstrated in similar South Asian populations (5). Since the TQP is based on parental report, it is likely to identify the more severe cases but miss more subtle or mild ones. Additionally, these results may be limited by recall bias, especially for the oldest children in our sample.

### Conclusions

Where past studies have found evidence of the increased risk of physical developmental delays associated with severe stunting, we also identified the risk of cognitive delay. It has been recommended for future work that a more sophisticated disability screen be developed which might include a greater number of questions and scaled categories for responses (28). Our study helps identify the specific disabilities that might be useful to target in developing a more specific screening tool. The contribution of our study is that it demonstrates the feasibility and use of the TQ tool in rural Nepal and confirms the association with nutritional status seen in other populations. We believe that this indicates an unmet need and hope that it will draw attention to the need to screen, definitely diagnose, and intervene to treat these disabilities.

## ACKNOWLEDGEMENTS

The study was conducted by the Department of International Health, Bloomberg School of Public Health, Johns Hopkins University (JHU), Baltimore, MD, USA, under grants from the National Institutes of Health, Bethesda, MD (HD 38753), the Bill & Melinda Gates Foundation, Seattle, Washington, DC (810-2054), and a Cooperative Agreement between the JHU and the Office of Health and Nutrition, US Agency for International Development, Washington, DC (HRN-A-00-97-00015-00).

The authors acknowledge Dr. Naila Khan, Head of the Child Development Centre, Child Development and Neurology Unit, Dhaka Shishu (Children's) Hospital, for her advice and training of the study investigators in the administration of the TQP. The authors have no financial relationships or competing interests relevant to this article to disclose. The corresponding and first authors had full access to all the data in the study and take responsibility for the integrity of the data and the accuracy of the data analysis.
